# An assessment of the outcomes of prevention of mother-to-child transmission of HIV services in Addis Ababa, Ethiopia

**DOI:** 10.4102/curationis.v39i1.1583

**Published:** 2016-05-11

**Authors:** Tefera G. Negash, Valerie J. Ehlers

**Affiliations:** 1 Department of Health Studies, University of South Africa, South Africa

## Abstract

**Background:**

This article assessed maternal and neonatal outcomes amongst users of prevention of mother-to-child transmission (PMTCT) of HIV services in Addis Ababa, Ethiopia.

**Objectives:**

The study aimed to assess the health outcomes (antiretroviral prophylaxis versus antiretroviral treatment, CD4 counts, World Health Organization (WHO) stages of illness, other illnesses) of women who had used these services, as well as the HIV status of their babies and the infant feeding method adopted.

**Methods:**

A quantitative, cross sectional, retrospective cohort design was used. Document reviews were conducted with a sample of 384 mother-infant pairs (out of a population of 796) who had used PMTCT services.

**Results:**

All respondents were using prophylactic antiretrovirals or antiretroviral therapy, but some were on the wrong treatment based on their CD4 counts. The CD4 counts increased four times more for women on antiretroviral treatment than for those on prophylactic antiretrovirals. The WHO’s stages of HIV illness did not improve but deteriorated in some cases, and 52 other illnesses were recorded. Out of the 384 infants, 6.0% (*n* = 23) were HIV-positive. Most respondents opted for exclusive breast feeding but some used mixed feeding during the first six months of their infants’ lives, despite having received health education related to infant feeding options.

**Conclusion:**

The respondents’ improved CD4 counts were inadequate to improve their World Health Organization stages of HIV illness. Some babies received mixed feeding during the first six months of their lives and 6% of the babies were HIV-positive despite their mothers’ utilisation of PMTCT services.

## Introduction

Prevention of mother-to-child transmission (PMTCT) services are available in Ethiopia, but during 2012 only an estimated 50% of pregnant women used these services (Joint United Nations Program on HIV/AIDS [UNAIDS] 2013:38–40), implying that 50% of these pregnant women did not access PMTCT services. Reasons for non-utilisation of PMTCT services were unknown, but might have been affected by the women’s lack of knowledge about the maternal and neonatal outcomes of the programme.

Since the 2001 declaration of commitment at the United Nations General Assembly Special Session (UNGASS) on Human Immuno Deficiency Virus/Auto Immune Deficiency Syndrome (HIV/AIDS), considerable progress has been made by PMTCT programmes. In 2009, 53% of HIV-positive pregnant women in low and middle income countries received antiretroviral medications (ARVs) for PMTCT. According to the World Health Organization ([WHO] 2011:1–12), four elements are helpful to reduce the incidence of PMTCT:

- Primary prevention of HIV infections.
- Family planning.
- Antiretroviral therapy (ART) interventions.
- Care, treatment and follow-up services.

The goal of PMTCT programmes is to maximise the health of the HIV-positive woman and decrease the chances of mother-to-child transmission of HIV (MTCT), by decreasing the viral load (VL) and increasing the CD4 count, whilst maintaining the maximum level of health throughout pregnancy (WHO 2011:1–12). Proper implementation of these four prongs of PMTCT services could reduce the incidence of MTCT and improve the quality of these services.

### Problem statement

PMCT services are provided in Addis Ababa, striving to enable the HIV-positive mothers to enjoy a good health-related quality of life and to enable at least 95% of their infants to be born and to remain HIV-negative (WHO 2010:3). However, the maternal and neonatal outcomes amongst the users of these PMTCT services were unknown, but should be known so that HIV-positive pregnant women could make informed decisions, based on PMTCT outcomes, about the utilisation of PMTCT services.

#### Aims of the study

This article aimed to determine the outcomes of PMTCT services related to maternal health and the infants’ HIV status and feeding method, in Addis Ababa. Based on the article’s potential findings, recommendations could be made to improve the maternal and neonatal outcomes amongst the users (mothers and neonates) of these services, by addressing identified shortcomings. If the PMTCT services have good maternal and neonatal outcomes, this information should be shared with the population of Addis Ababa, possibly motivating more pregnant women to utilise PMTCT services.

#### Background information

As more HIV-positive women live longer and enjoy a better health-related quality of life because of ART, more and more women will require PMTCT services (Central Statistical Agency of Ethiopia & Inner City Fund International [CSAE/ICFI] 2012:232–236), making on-going PMTCT-related research essential to continue improving the outcomes of these services.

PMTCT services should be utilised by most (preferably by all) pregnant women, whose health should benefit and whose babies should be HIV-negative (WHO 2010:3).

#### Research objectives

The objectives of this study were to:

- Assess the health outcomes (CD4 counts, WHO stage of illness, other illnesses) of women who had used PMTCT services in Addis Ababa.
- Compare the maternal outcomes of HIV-positive pregnant women who used prophylactic ARVs compared to those who used ART.
- Assess the outcomes (HIV status and infant feeding method) of babies whose mothers had utilised PMTCT services.

#### Definitions of key concepts

Antiretroviral therapy (ART) implies the use of ARV drugs to restore immune function, maintain maximum suppression of viral replication, reduce HIV-related morbidity and mortality and improve the quality of life with prolonged survival rates for both mothers and babies (Ethiopian Federal Ministry of Health (EFMOH 2008:48). Antiretrovirals (ARVs) for PMTCT include the administration of ARVs to HIV-positive mothers and their infants to prevent MTCT. This includes ART provision for those pregnant women who were eligible for treatment and temporary ARV prophylaxis for those who were not eligible for ART, based on their CD4 counts.

Human Immunodeficiency Virus (HIV) is a virus that damages the body’s immune system, the system that fights infections (Longo & Fauci 2005:1071–1075). HIV is an aetiologic agent of AIDS and can be transmitted by sexual contact, from blood and blood products, sharing injection needles or instruments used in tattooing or circumcision and by HIV-infected mothers to their infants during pregnancy, birth or breast feeding (Fauci & Lane 2005:1076–1139).

PMTCT refers to the reduction of new paediatric HIV infections through the promotion of primary prevention of HIV amongst women and men of reproductive age, addressing family planning within the context of HIV, promoting access to HIV and ART or providing prophylactic treatment for HIV-infected pregnant women and their families and promoting access of HIV-exposed infants to care (EFMOH 2011:3). This article focused on the following PMTCT aspects: maternal outcomes (CD4 counts, WHO stage of HIV illness and other illnesses compared for women using prophylactic ARVs and those using ART) and neonatal outcomes (infants’ HIV status and infant feeding practices).

#### Contribution to the field of study

The findings from the article could inform public health professionals at different levels about the maternal and neonatal outcomes of the PMTCT programme (basically implying healthy mothers and HIV-negative babies). This information could help to enhance programme management, improve PMTCT services, policy development and implementation as well as further research.

### Literature review

During 2009, an estimated 2.6 million people were newly HIV-infected worldwide. In sub-Saharan Africa (SSA), 1.8 million people became HIV-infected during the same year (UNAIDS 2013:4). The annual number of AIDS-related deaths decreased from 2.1 million in 2004 to 1.8 million in 2009. This trend is attributable to the increased expansion of ART programmes, including PMTCT services (UNAIDS 2010:16–30). The number of people living with HIV in 2012 was 35.3 million. During the same year, 2.3 million people were newly infected with HIV globally, a 33% decline from 2001. At that time 1.6 million people died from HIV, compared to 2.3 million in 2005 (UNAIDS 2013:4). During 2012 the reported HIV prevalence in Ethiopia was 1.5% amongst adults aged 15 to 49 years (1.0% amongst men and 1.9% amongst women). However, the prevalence in Addis Ababa was 5.2% (4.3% amongst men and 6.0% amongst women). The HIV prevalence amongst women attending antenatal care (ANC) clinics from 2008 to 2011 in the public sector was 1.7% whilst it was 3.1% outside the public sector (CSAE/ICFI 2012:232–236). As Addis Ababa has a higher HIV prevalence than Ethiopia’s national HIV prevalence, HIV services are provided throughout this area, rendering it a suitable area for conducting research about the outcomes of PMTCT services.

PMTCT services prevented more than 670 000 children from becoming infected with HIV from 2009 to 2012 (UNAIDS 2013:38–40). However, Ethiopia was amongst the countries with less than 50% utilisation of PMTCT services during 2012 (UNAIDS 2013:38–40).

Preventing HIV from infecting infants during breast feeding should balance protection from other causes of morbidity and mortality. The WHO recommends that mothers should continue breast feeding and use ARVs or avoid breast feeding altogether. This decision is affected by the socio-economic and cultural context of the population, availability and quality of health services, main causes of maternal and child under-nutrition and infant and child mortality rates. It is also necessary to inform HIV-infected mothers about the available alternatives to breast feeding: ‘… without causing harm to the general population’s infant feeding practices’ (WHO 2010:3).

Belachew and Jira (2007:41–42) reported that out of 657 respondents, 60% mentioned exclusive replacement feeding for infants younger than six months of age as an option for preventing MTCT in the Gurage zone of Ethiopia whilst 24% mentioned breast milk and 16% mentioned mixed feeding but they did not know what to feed their infants. Only 24% knew that MTCT could occur through breast feeding. More information was required to increase mothers’ infant feeding knowledge and to improve the outcomes of the PMTCT programme (WHO 2010, 2011).

## Research method and design

### Design

A quantitative, cross sectional, retrospective, descriptive cohort design was used. A cohort was assembled amongst women and their children who used PMTCT services at hospitals and health centers in Addis Ababa within two years prior to the study.

### Population and sample

Initially the study sites were randomly selected, stratified according to the three major types of facilities providing PMTCT services in Addis Ababa, namely public hospitals, private hospitals and public health centers. (Public hospitals and public health centers are funded by Ethiopia’s government and provide free health care services whilst private hospitals can charge fees-for-services-rendered and/or receive subsidies from the Ethiopian government for rendering specific services). Thereafter the respondents were randomly selected from each site in proportion to the number of patients who used PMTCT services at the site concerned.

### Selection of study sites

In 2012 PMTCT services were provided by 61 hospitals and health centers in Addis Ababa, comprising the site target population for this study. The sites comprised 10 public hospitals, 15 private hospitals and 36 public health centers. Proportionate stratified random sampling was used to select specific health care facilities within each stratum. The names of all hospitals and health centers providing PMTCT services were placed on folded slips of paper in separate containers representing the respective strata of public hospitals, private hospitals and public health centers. A person randomly drew two names from the public hospitals, three from the private hospitals and seven from public health centers. These 12 sites comprised the stratified randomly selected site sample for the current study.

### Selection of respondents

The target population comprised 796 mother-infant pairs who used PMTCT services at the 12 facilities participating in this study during 2013. The inclusion criteria required mother-infant pairs to have used PMTCT services, infants’ HIV test results should be known and the mothers should have signed consent for themselves and on behalf of their babies. Exclusion criteria were mother-infant pairs who discontinued PMTCT services, pregnant women at the time of data collection, babies whose HIV test results were unknown and those who used PMTCT services at facilities that did not participate in this study, and mothers who did not sign. Patients’ ART record numbers were used. Then a table of random number was used to select women attending PMTCT services. The number of mother-infant pairs selected was proportional to the total number of patients treated at each participating PMTCT hospital or health center.

The formula used for sample size calculation of respondents was *S* = *p* (1-*p*) *z*^2^/*d*^2^ where *p* stands for anticipated population proportion, *z* refers to the cut-off value of the normal distribution and *d* is the precision required on either side of the proportion (Sayed 2007:347), amounting to a sample size of 384; but 19 additional respondents were selected so that they could participate in pre-testing of the checklist. It was assumed that with a 95.0% confidence interval, 80% of the women who used PMTCT services would have improved health status and 5% would be the worst acceptable result.

### Materials

Document review checklists were used to capture data from the mothers’ and from the babies’ files. The information gathered included personal characteristics (such as the mother’s age, marital and education status), whether the mother used ARV prophylaxis or ART, maternal outcomes (CD4 count, WHO stage of HIV disease and other illnesses) and neonatal outcomes (neonatal HIV status and feeding practices).

Pre-testing of the checklist was carried out by three trained research assistants who recorded 19 mother-infant pairs’ information on the checklists from their files. The information obtained during the pre-test phase was not used during the data analysis. There was no need to implement any alterations on the checklist based on the pre-test’s results. (The 19 maternal-neonatal pairs of records used during the pre-test phase were obtained from the stratified randomly selected respondents as 19 additional respondents had been selected to accommodate the pre-testing of the checklist.)

### Data collection procedures

All three research assistants were nurses who were fluent both in English and Amharic. They recorded data from patients’ records in English on the checklists. The first author supervised the data collection procedures and could provide assistance if and when necessary. The first author also conducted ‘spot checks’ comparing the recorded information with the information on selected patients’ files. The actual data collection took place from May to November 2013.

### Data analysis

Both descriptive and inferential statistics, such as the mean and chi-square (*X*^2^) tests, were used to analyse the data. A *p*-value of 0.05 was used as a cut-off value for significant associations (between maternal outcomes CD4 counts, WHO stage of HIV illness and other illnesses compared for women using prophylactic ARVs and those using ART) and neonatal outcomes (namely infants’ HIV status and infant feeding practices).

At a confidence interval of 95%, each variable was analysed using descriptive statistics before associations were calculated. Standard deviations and measures of central tendency (mean, median and mode) were used for ARV intervention and infant feeding practices and counselling. A statistician assisted with the data analysis and interpretations.

The Statistical Package for the Social Sciences (SPSS version 20.0) was used for data analysis. Epi Info version 3.5.1 was used for calculating statistics that could not be calculated by SPSS. Microsoft Excel 2007 was used for plotting graphs.

## Results

### Biographic information

The average age of the respondents was 28.1 years, ranging from 19 to 38 years, with the modal age group being 26 to 30 years (46.9%; *n* = 180). Most respondents (88.5%; *n* = 340) were married. Although 37.7% (*n* = 145) of the respondents had completed grades 9–12, 24.7% (*n* = 95) completed grades 7–8, 5.7% (*n* = 22) never attended school, 17.2% (*n* = 66) only managed to attend grades 1 to 6 at school whilst 14.6% (*n* = 56) had attended universities/colleges.

### Prophylactive antiretrovirals (ARVs) versus antiretroviral treatment (ART)

Out of 384 respondents, 14% (*n* = 55) received ARV prophylaxis and 86% (*n* = 329) received ART for PMTCT. Out of the 55 respondents on ARV prophylaxis, 25.5% (*n* = 14) commenced this regimen at CD4 counts of < 350 cells/mm^3^, when they were supposed to receive ART. Conversely, out of 329 women on ART, 20.4% (*n* = 67) had CD4 counts of ≥ 350 cells/mm^3^, implying that they were supposed to receive ARV prophylaxis.

### CD4 counts

All respondents (100%; *n* = 384), had CD4 counts recorded, ranging from one to six times. The respondents’ mean CD4 counts increased from 302.1 cells/mm^3^ to 414 cells/mm^3^, being a significant finding according to the repeated measure analysis of variance (ANOVA) at 0.05 *p*-value (*p* = 0.008).

Amongst those women who received ART, the mean CD4 count increased from 267 cells/mm^3^ to 329 cells/mm^3^. Therefore the increment of CD4 counts amongst women on ART was four times higher than that of women on ARV prophylaxis. Correlation analysis showed that a higher CD4 count at commencement of ARVs, correlated with a higher most recent CD4 count, whilst a lower CD4 count at commencement of ARVs correlated with a lower most recent decreased CD4 count. This was statistically significant at 0.05 *p*-value (*r* = 0.613; *p* < 0.01). Hence, the following linear regression model predicted that when the initial CD4 increased by 1 cell/mm^3^, the most recent CD4 count increased by 0.665 cells/mm^3^.

Most recent CD4 count = 154 + 0.665 (CD4 count when ARVs commenced).

### World Health Organization stages of HIV illness

The recorded WHO stages of HIV illness decreased from 100% (*n* = 384) at the first to 31.3% (*n* = 120) at the sixth ANC visit (as shown in Table 1), indicating that the records of the PMTCT programme should be improved in future.

**TABLE 1:** Maternal World Health Organization HIV stage of illness at six different times.

WHO staging at six different times	Percent and number of women in each WHO stage				Total % (*n*)

	WHO stage I % (*n*)	WHO stage II % (*n*)	WHO stage III % (*n*)	WHO stage IV % (*n*)	
First time WHO staging	21.4 (82)	53.9 (207)	23.7 (91)	1 (4)	100 (384)
Second time WHO staging	19.4 (69)	56.1 (199)	23.4 (83)	1.1 (4)	100 (355)
Third time WHO staging	17.6 (59)	56.7 (190)	24.2 (81)	1.5 (5)	100 (335)
Fourth time WHO stating	28.0 (51)	41.8 (76)	26.9 (49)	3.3 (6)	100 (182)
Fifth time WHO staging	32.1 (42)	35.1 (46)	28.2 (37)	4.6 (6)	100 (131)
Sixth time WHO staging	30.8 (37)	35.8 (43)	29.2 (35)	4.2 (5)	100 (120)

*Source*: Negash, T.G., 2014, ‘Review of prevention of mother-to-child transmission of HIV in Addis Ababa, Ethiopia’, DLit et Phil thesis, University of South Africa, Pretoria

Concerning the WHO stages of HIV illness (WHO 2005), out of 384 respondents, 19.8% (*n* = 76) were in WHO stage I, 54.2% (*n* = 208) in WHO stage II, 24.2% (*n* = 93) in WHO stage III and 1.8% (*n* = 7) in WHO stage IV when ARVs were commenced. Out of the respondents in WHO stage I, 36 received ARV prophylaxis and 40 received ART. Out of 208 patients in WHO stage II, 15 received ARV prophylaxis and 193 received ART whilst out of those in WHO stage III, four received ARV prophylaxis and 89 received ART. All patients in WHO stage IV, received ART.

During the initial visit, 53.9% (*n* = 207) of the respondents were in WHO stage II but this percentage decreased as their conditions deteriorated causing increased percentages in WHO stages III and IV. Hence, maternal health status, in terms of WHO staging, showed no improvement, indicating that the CD4 count did not increase sufficiently to maintain or improve their clinical health status, as shown in Table 2.

**TABLE 2:** CD4 counts and WHO stages of HIV illness when antiretrovirals were commenced (*n* = 384).

Variables	ARV Prophylaxis % (*n*)	ART % (*n*)	Total % (*n*)
**CD4 count at ARV commencement**			
< 350 cells/mm^3^	5.1 (14)	94.9 (262)	100 (276)
≥ 350 cells/mm^3^	38 (41)	62 (67)	100 (108)
**WHO stage at ARV commencement**			
WHO stage I	47.4 (36)	52.6 (40)	100 (76)
WHO stage II	7.2 (15)	92.8 (193)	100 (208)
WHO stage III	4.3 (4)	95.7 (89)	100 (93)
WHO stage IV	0.0 (0)	100 (7)	100 (7)

**Total**	**14.3 (55)**	**85.7 (329)**	**100 (384)**

*Source*: Negash, T.G., 2014, ‘Review of prevention of mother-to-child transmission of HIV in Addis Ababa, Ethiopia’, DLit et Phil thesis, University of South Africa, Pretoria

ARV, antiretrovirals; ARV Prophylaxis, antiretroviral Prophylaxis; ART, antiretroviral therapy.

### Other maternal illnesses

The figures in Table 3 indicate that at enrolment into the PMTCT programme, 34 illnesses were diagnosed and 19 more during follow-up visits, totalling 53 illnesses. As indicated in Table 3, the most prevalent illnesses at enrolment were oral candidiasis (23.2%; *n* = 89) followed by pulmonary tuberculosis (10.2%; *n* = 39). The most prevalent illness during follow-up visits was urinary tract infection (6.5%; *n* = 25), followed by upper respiratory tract infections (5.8%; *n* = 22) and acute gastroenteritis (3.4%; *n* = 13).

**TABLE 3:** Maternal illnesses most frequently diagnosed at enrolment and during the follow-up phases of Prevention of mother-to-child transmission services.

S.No.	Illnesses at enrolment into PMTCT service	Illnesses during follow-up of PMTCT service
1	Oral Candidiasis (*n* = 89)	UTI (*n* = 25)
2	Pulmonary TB (*n* = 39)	URTI (*n* = 22)
3	URTI (*n* = 39)	Pneumonia (*n* = 20)
4	Herpes Zoster (*n* = 34)	Herpes Zoster (*n* = 20)
5	Pneumonia (*n* = 23)	Acute Gastroenteritis (*n* = 13)
6	Seborrhoea (*n* = 12)	Oral Candidiasis (*n* = 13)
7	Cellulitis (*n* = 10)	Diarrhoea (*n* = 7)
8	Prurigo (*n* = 9)	Acute Bronchitis (*n* = 7)
9	UTI (*n* = 7)	Tonsillitis (*n* = 6)
10	Oral Hairy Leukoplakia (*n* = 6)	Anaemia (*n* = 4)
11	Diarrhoea (*n* = 6)	Cellulitis (*n* = 4)
12	Angular Cheilitis (*n* = 6)	Typhoid Fever (*n* = 4)

*Source*: Negash, T.G., 2014, ‘Review of prevention of mother-to-child transmission of HIV in Addis Ababa, Ethiopia’, DLit et Phil thesis, University of South Africa, Pretoria

PMTCT, Prevention of mother-to-child transmission; UTI, Urinary tract infection; URTI, Upper respiratory tract infections; Pulmonary TB, Pulmonary tuberculosis.

### Babies’ HIV status

Out of the 384 babies, 6.0% (*n* = 23) tested HIV-positive, implying that 94.0% (*f =* 361) were HIV-negative.

### Infant feeding practices

As displayed in Table 4, on average a respondent had been counselled 3.46 times by health care providers about infant feeding. Of these respondents, 83% (*n* = 319) had been counselled on exclusive breast feeding during the first six months, 11% (*n* = 42) on mixed feeding and 5.5% (*n* = 21) on replacement feeding. Most women (87%; *n* = 334) practiced exclusive breast feeding during the first six months, 2.9% (*n* = 11) used mixed feeding and 10.2% (*n* = 39) replacement feeding.

**TABLE 4:** Infant feeding practices (*n* = 384).

Infant feeding counselling/practice	Exclusive breast feeding % (*n*)	Mixed feeding % (*n*)	Replacement feeding % (*n*)	None recorded % (*n*)	Total % (*n*)
Infant feeding counselling	83 (319)	11 (42)	5.5 (21)	0.5 (2)	100 (384)
Infant feeding practice	87 (334)	3 (11)	10 (39)	0 (0)	100 (384)

*Source*: Negash, T.G., 2014, ‘Review of prevention of mother-to-child transmission of HIV in Addis Ababa, Ethiopia’, DLit et Phil thesis, University of South Africa, Pretoria

## Ethical considerations

All ethical principles were respected such as autonomy, anonymity, confidentiality, privacy, justice and beneficence. Ethical clearance was granted by the University of South Africa’s Higher Degrees Committee and by the Addis Ababa City Administration Health Bureau Ethical Clearance Committee. The heads of all 12 participating hospitals and health centers granted permission to collect data at their facilities.

### Potential benefits and hazards

Respondents received no remuneration and no direct benefits, but they and other pregnant women might benefit from potentially improved PMTCT services in future, based on some of the article’s findings. No one was exposed to any hazard as information was gathered by conducting structured interviews and from the respondents’ and infants’ medical files.

### Recruitment procedures

From each selected site, the respondents were selected using simple random sampling to ensure representativeness. A table of random numbers was used to select women attending PMTCT services. Those selected women were contacted by the research assistants during their follow-up visits to the PMTCT service sites when they were requested to sign consent that their own and their babies’ medical files could be used to obtain data for the study. The number of mother-infant pairs selected was proportional to the number of patients treated at each health facility.

### Informed consent

Every randomly selected woman was informed about the purpose and nature of the study. She was asked to give consent that information from her and from her baby’s files could be used anonymously. Every woman was reassured that her decision to participate in the study or not would not affect the services rendered to her and her baby in any manner whatsoever.

### Data protection

No names were used on any checklist. The researcher kept a list linking each mother’s number with her medical file’s number and with her infant’s file number, so that correlations could be calculated and an audit trail would be available in case queries arose about the study’s findings. Only the first author had access to this list which was kept under lock and key and which would be destroyed after acceptance of the research report. The research assistants signed confidentiality agreements with the first author.

### Reliability

Prior to data collection, the document review checklist was pre-tested and revised based on identified shortcomings. To ensure internal reliability Chronbach’s alpha coefficients were calculated and found to be 0.7. A Cronbach alpha of 0.7 was regarded as being acceptable (Burns & Grove 2005:374).

### Validity

The tool was tested for face, content and construct validity. For content validity, four experts working in PMTCT services conducting PMTCT research were consulted to ensure that every item in the data collection tool related to PMTCT services. For face validity these experts had to agree that the checklist appeared to request information about the maternal and neonatal outcomes of the PMTCT programme. An appropriately defined population, a large sample size and a proportional stratified random sampling technique enhanced the external validity of the study, implying that the findings of this study could be generalised to the population of mother-infant pairs using PMTCT services in Addis Ababa.

## Discussion

### Biographic data

These findings imply that 22.9% (*n* = 88) of the respondents’ highest school levels ranged from no schooling up to grade 6. Women with such limited education might find it difficult to comprehend the complex nature of HIV and PMTCT. However, according to Ismail and Ali (2011:129–130), 91.4% of the Ethiopian women who participated in their study, understood the health education information and 89.8% were satisfied with HIV-related counselling and discussions provided at clinics.

As the respondents’ average age was 28.1 years, they could have a number of future pregnancies requiring up-to-date knowledge about the prevention of HIV re-infection, about PMTCT and about contraception. Most respondents (88.5%; *n* = 340) were married. Consequently, HCT and PMTCT services for couples might of benefit to these women.

### Prophylactic antiretrovirals (ARVs) versus antiretroviral treatment (ART)

Most (86.0%; *n* = 329) of the current study’s respondents used ART whilst only 14.0% (*n* = 55) used prophylactic ARVs. Some women who should have received ART were on prophylactic ARVs and vice versa. The data for the current study were collected prior to the implementation of WHO option B (providing lifelong ART to all pregnant women as part of PMTCT) care which should avoid such errors in future (WHO 2013). The CD4 increases for women on ART were four times higher than those for women on prophylactic ARVs, indicating that women who had erroneously received prophylactic ARVs could have suffered negative consequences, and possibly also their babies, but this could not be confirmed or denied based on the article’s available data. Nevertheless, the revised WHO (2013) guidelines requiring all HIV-positive pregnant women to receive ART would have greater benefits than merely administering prophylactic ARVs to women with higher CD4 counts.

### CD4 counts

The CD4 counts of all respondents improved significantly, but the average increased CD4 count for women on ART was significantly higher than for women on prophylactic ARVs. Women with higher CD4 counts when ARVs or ART were commenced, had higher CD4 increases compared to women with lower CD4 counts at the commencement of both forms of treatment. A Kenyan study supports this finding because women with CD4 counts of < 250 cells/mm^3^ decreased from 23% at baseline to 5% at 24 weeks postpartum (Okonji *et al*. 2012:251–254). Thus, findings from the article, as well as from Okonji *et al*.’s (2012–252) study, support the WHO (2013) guideline of administering ART to all HIV-positive pregnant women, irrespective of their CD4 count to produce improved PMTCT outcomes related to CD4 counts. A study from South Africa reported similar findings, indicating that only 1.51% of the 1369 women in their study progressed from a high antenatal to a low postnatal CD4 count (Lebon *et al*. 2007:1472–1473) if they used ART.

### World Health Organization stages of illness

Although the CD4 counts of all respondents increased, the WHO stages of illness showed no improvement. The percentage of respondents in WHO stage II decreased as their condition deteriorated and some progressed to stages III and IV. Thus, the increase in the CD4 counts might have been inadequate to enable the women’s clinical conditions to remain stabilised or to improve. Findings from a multi-country study in Africa reported that 4.4% of women developed new WHO stage III and 0.4% of them developed new WHO stage IV during their pregnancies, supporting the current study’s findings (Ekouevi *et al*. 2012:4–6). A multi-country study in Africa used CD4 count and WHO stage to measure maternal health status. The finding related to CD4 count was contrary to the article’s finding but the WHO stage finding supported its finding. At 12 and 24 months after their babies’ births, 4.5% and 11.6% of women with enrolment CD4 count of > 250 cells/mm^3^ had CD4 decline to < 200 cells/mm^3^. A total of 4.4% of women developed new WHO stage III and 0.4% of them developed new WHO stage IV (Ekouevi *et al*. 2012:4–6) despite using PMTCT services.

### Other illnesses

Although a total of 53 illnesses were diagnosed amongst the current study’s respondents, they responded to treatment. A study from the USA examined the association of latest CD4 counts with risk of non-AIDS diseases in a cohort of 1397 patients who initiated ART. A total of 80 patients developed non-AIDS events. However, higher CD4 counts (amongst patients using ART) were associated with lower rates of non-AIDS events (Baker *et al*. 2008:841).

### Babies’ HIV status

By the time infants were ≤ 6 weeks of age, 65.6% (*n* *=* 253) of them had been tested with the DNA-PCR test and when they were > 6 weeks of age 34.4% (*n* *=* 132) of them had been tested. Consequently these infants’ HIV status could not be categorised in terms of prenatal or postnatal MTCT. Nevertheless the crude HIV infection rate amongst the 384 babies was 6.0% (*n* = 23). This was slightly higher than the infection rate of 5.0% for effective PMTCT progammes, in terms of the WHO’s recommendations (WHO 2013).

The factors that had significant effects on the rate of MTCT included nipple fissures, ARV prophylaxis taken by the mother versus ART, ARV prophylaxis given to the infant, birth weight, gestational age during delivery and APGAR score. However, gender of the infant, mode of delivery and maternal age did not have any significant associations with the rate of MTCT. As HIV status of the infant is a dichotomous categorical outcome variable (HIV-positive versus HIV-negative), binary logistic regression was used. The logistic regression model predicted correctly that 96% of the infants would be HIV-negative;

$$Y=-25.89+0.05(X_{1})+22.64(X_{2})+0.45(X_{3})+1.76(X_{4})+2.46(X_{5})+0.33(X_{6})$$ [Eqn 1]

Symbols *Y* and *X* used to represent the dependent and independent variables as:

- *Y* = HIV status of the infant.
- *X*_1_ = Nipple fissure.
- *X*_2_ = ARVs given for the baby.
- *X*_3_ = Newborn weight.
- *X*_4_ = Gestational-age-at-delivery.
- *X*_5_ = APGAR score.
- *X*_6_ = ARVs given for the mother.

Infants whose mothers had nipple fissures were 20 times more likely to be infected with HIV as compared to infants born from mothers without nipple fissures. Infants who did not take ARV prophylaxis were 23 times more likely to be infected with HIV as compared to those who received ARV prophylaxis.

When birth weight of the infant increases by 1 kg from low birth weight to normal birth weight, the odds of an infant free from HIV increases by a factor of 0.45. After an increase in one week’s gestational age at birth, the odds of an infant free from HIV increases by a factor of 1.76. The model predicts that with an increase in APGAR score by 1, the odds of an infant free from HIV increases by a factor of 2.46. Infants born from mothers who took ARV prophylaxis were three times more likely to be infected with HIV as compared to those infants born from mothers who took ART. Table 5 portrays the logistic regression of factors affecting the MTCT rate amongst HIV-positive mothers’ infants.

**TABLE 5:** Logistic regression: Factors affecting mother-to-child transmission.

Variables in the equation		B	S.E.	Wald	*df*	Sig.	Exp (B)
Step 1	Nipple fissure (1)	−3.009	0.636	22.411	1	0.000	0.049
	Baby-ARV-prophylaxis (1)	3.120	1.200	6.758	1	0.009	22.636
	Newborn weight	−0.806	0.392	4.213	1	0.040	0.447
	Gestational-age-at-delivery	0.564	0.145	15.109	1	0.000	1.758
	APGAR-score	0.898	0.382	5.545	1	0.019	2.456
	ARVs-of-the-mother (1)	−1.118	0.683	2.677	1	0.102	0.327
	Constant	−25.887	7.382	12.298	1	0.000	0.000

*Source*: Negash, T.G., 2014, ‘Review of prevention of mother-to-child transmission of HIV in Addis Ababa, Ethiopia’, DLit et Phil thesis, University of South Africa, Pretoria

Variable(s) entered on step 1: nipple fissure, baby’s-ARV-prophylaxis, newborn weight, gestational-age-at-delivery, APGAR-score, ARVs/ART-of-the-mother.

MTCT, mother-to-child transmission.

Out of the 384 mothers, 6.8% (*n* = 26) had nipple fissures and 38.5% of their infants were HIV-positive whilst 3.6% of infants whose mothers did not have nipple fissures were HIV-positive. Fisher’s exact test also shows a significant association between infants’ HIV-positive status and nipple fissures at 0.05 *p*-value (*p* < 0.01), as shown in Tables 6 and 7.

**TABLE 6:** Fisher’s exact test of HIV status versus nipple fissure.

Variables	Value	*df*	Asymp. Sig. (2-sided)	Exact Sig. (2-sided)	Exact Sig. (1-sided)
Fisher’s exact test	-	-	-	0.000	0.000
Number of valid cases	384	-	-	-	-

*Source*: Negash, T.G., 2014, ‘Review of prevention of mother-to-child transmission of HIV in Addis Ababa, Ethiopia’, DLit et Phil thesis, University of South Africa, Pretoria

**TABLE 7:** HIV status versus nipple fissure (*n* = 384).

Nipple fissure	HIV status		Total % (*n*)

	HIV-positive % (*n*)	HIV-negative % (*n*)	
Present	38.5 (10)	61.5 (16)	100 (26)
Absent	3.6 (13)	96.4 (345)	100 (358)
**Total**	**6 (23)**	**94 (361)**	**100 (384)**

*Source*: Negash, T.G., 2014, ‘Review of prevention of mother-to-child transmission of HIV in Addis Ababa, Ethiopia’, DLit et Phil thesis, University of South Africa, Pretoria

### Infant feeding practices

Although most (83.0%; *n* = 319) respondents used exclusive breast feeding until the babies were six months old, some (11.0%; *n* = 42) used mixed feeding. No reasons were provided for choosing mixed feeding at such an early age, but it is known that Ethiopian mothers give mixed feeds to their infants from a very early age (Belachew & Jira 2007:40).

## Limitations of the study

Only mother-infant pairs who had used PMTCT services could be considered for participation in this study. HIV-positive women who did not use, or who discontinued using PMTCT services, might have had different views, but they were not interviewed because they could not be accessed.

Although the first author conducted spot checks comparing information recorded on checklists with selected patients’ PMTCT records, no systematic audits of all these records could be conducted, and no observations of actual provider-patient interactions were recorded. Such systematic audits and observations might have revealed aspects of PMTCT services that require improvements in Addis Ababa.

As CD4 counts and WHO disease stages were not recorded for every woman at every ANC visit, the conclusions based on the available information might be inaccurate and should be regarded as indicating a statistical trend, rather than factual statistics.

No interviews were conducted with the health care providers. Consequently their perspectives remain unknown.

Viral loads were not carried out routinely during the data collection phase and were thus unavailable for compiling correlations with other variables such as CD4 counts and WHO stages of illness.

## Recommendations

### Recommendations for clinical practice

- Health education related to HIV and PMTCT should be appropriate for each woman’s educational status, as the respondents’ qualifications ranged from no schooling up to university/college education.
- PMTCT services in Ethiopia remain important because the average age of the 384 respondents was 28.1 years. Contraception services should thus form a vital part of PMTCT services.
- Voluntary counselling and testing for couples and PMTCT services focusing on married couples, rather than only on the HIV-positive pregnant women, should be considered as more than 80% of the current study’s respondents were married.
- Implementing the WHO’s option B (administering lifelong ART to all HIV-positive pregnant women) could have advantages for these women and for their babies.
- Nurses/midwives should ensure that every woman’s CD4 count and WHO illness stage are recorded at every ANC visit.
- Future records of PMTCT services should correlate the occurrence of any non-AIDS condition with the HIV-positive pregnant woman’s CD4 count and WHO stage of illness and with the baby’s HIV status.
- Counselling about infant feeding options should emphasise exclusive breast feeding during the first six months and the avoidance of mixed feeding during this time.
- ANC should ensure that as many babies as possible are born at full term, of normal weight and with good APGAR scores to reduce their chances of being born HIV-positive.
- Mothers with nipple fissures should refrain from breastfeeding their babies as these babies had a 20 times greater risk of being HIV-positive than their counterparts whose mothers did not have nipple fissures.
- Babies should be given prophylactic ARVs as these babies’ risk of being HIV-positive was 23 times smaller than babies who did not receive prophylactic ARVs.

### Recommendations for future research

- Future research should identify reasons why pregnant women do not use and/or discontinue using PMTCT services by interviewing pregnant women who failed to use, or who discontinued using, PMTCT services.
- Records should be kept at all ANC clinics of HIV-positive pregnant women, and those who do not use PMTCT services should be followed up by health care providers. Detailed records should be kept for comparing maternal and neonatal outcomes of HIV-positive pregnant women who utilised PMTCT services compared to those who did not.
- Qualitative research should gather more data related to HIV-positive women’s experiences of utilising PMTCT services, and to the perceptions of women who fail to use these services. Further research is also recommended to assess why women with improved CD4 counts did not have improved clinical conditions.
- Regular audits should be carried out on patients’ records, shortcomings identified and addressed. If patients’ viral loads are determined in future, regular comparisons of VL, CD4 count, WHO illness stage, and the occurrence of any illness should be correlated. These correlations should then be compared with the baby’s HIV status.
- Nurses/midwives’ experiences about providing PMTCT services should be studied quantitatively and qualitatively.

## Conclusions

All respondents’ CD4 counts improved, but the WHO illness stages showed no improvement. Women on ART had markedly increased CD4 counts compared to those who used prophylactic ARVs. Women with higher CD4 counts when they commenced taking ARVs, had higher CD4 counts after their babies’ births. Thus the WHO (2013) revised guidelines advocating ART for all pregnant HIV-positive women, irrespective of their CD4 counts, seem to be justified for producing enhanced maternal outcomes, based on the current study’s findings.

Effective antenatal as well as postnatal services are vitally important parts of the PMTCT programme because:

- HIV-positive mothers’ newborn babies of normal weight, full term gestation and high APGAR scores had better chances of being HIV-negative.
- Babies whose HIV-positive mothers had nipple fissures had a 20 times greater chance of being HIV-positive than their counterparts whose mothers had no nipple fissures.
- Babies who received prophylactic ARVs had their chances of being HIV-positive reduced 23 times.

The recommendations based on the article’s findings could help to improve Ethiopia’s PMTCT programme, update existing guidelines, update policies and conduct further research based on these findings. Therefore PMTCT programme managers, health care providers, health science students, researchers and HIV/AIDS patients could benefit from the findings of this study.

## References

[CIT0001] Baker J.V., Peng G., Rapkina J., Abrams D.I., Silverberg M.J., MacArthur R.D. et al, 2008, ‘CD4 count and risk of non-AIDS diseases following initial treatment for HIV infection’, *AIDS* 22(7), 841–848. 10.1097/QAD.0b013e3282f7cb76 18427202 PMC3618460

[CIT0002] Belachew T. & Jira C, 2007, ‘Awareness about feeding options for infants born to HIV positive mothers and mother to child transmission of HIV in Gurage zone, South Ethiopia’, *Ethiopian Journal of Health Development* 21(1), 40–47. 10.4314/ejhd.v21i1.10030

[CIT0003] Burns N. & Grove S.K, 2005, *The practice of nursing research: Conduct, critique and utilization*, 6th edn, W.B. Saunders, Philadelphia, PA.

[CIT0004] Central Statistical Agency of Ethiopia & Inner City Fund International (CSA/ICFI), 2012, *Ethiopia demographic and health survey 2011*, viewed 16 April 2012, from http://www.measuredhs.com/pubs/pdf/FR255/FR255.pdf

[CIT0005] Ekouevi D.K., Stringer E., Coetzee D., Tih P., Creek P., Stinson K. et al, 2012, ‘Health facility characteristics and their relationship to coverage of PMTCT of HIV services across four African countries’, *PLoS One* 7(1), 1–7. 10.1371/journal.pone.0029823 PMC3262794 22276130

[CIT0006] Ethiopian Federal Ministry of Health (EFMOH), 2008, *Guidelines for paediatric HIV/AIDS care and treatment in Ethiopia*, HIV/AIDS Prevention and Control Office, Addis Ababa.

[CIT0007] Ethiopian Federal Ministry of Health (EFMOH), 2011, *Guideline for prevention of mother-to-child transmission of HIV in Ethiopia*, viewed 27 November 2012, from http://www.etharc.org/resources/download/finish/59/707

[CIT0008] Fauci A.S. & Lane H.C, 2005, ‘The human deficiency virus disease: Aids and related disorders’, in Kasper D.L., Faucy A.S., Longo D.L., Braunwald E., Hausler S.L. & Jameson S.L. (eds.), *Harrison’s principles of internal medicine*, 16th edn, pp. 1076–1139, McGraw-Hill, New York.

[CIT0009] Ismail H. & Ali A, 2011, ‘Pregnant women’s satisfaction and comprehension level of information given during HIV counseling and testing for PMTCT in public health facilities in Addis Ababa’, *Ethiopian Journal of Health Development* 25(2), 126–134.

[CIT0010] Joint United Nations Progamme on HIV/AIDS (UNAIDS), 2010, ‘*Global report on AIDS epidemic*’, viewed 12 April 2012, from http://www.unaids.org

[CIT0011] Joint United Nations Progamme on HIV/AIDS (UNAIDS), 2013, ‘*Global report on AIDS epidemic*’, viewed 12 April 2012, from http://www.unaids.org

[CIT0012] Lebon A, Bland R.M, Rollins N.C, Coutsoudis A, Coovadia H & Newell M.L, 2007, Short communication: CD4 counts of HIV-infected pregnant women and their infected children-implication for PMTCT and treatment programmes’, *Tropical Medicine and International Health*, 12(12), 1472–1474. 18076554 10.1111/j.1365-3156.2007.01954.x

[CIT0013] Longo D.L. & Fauci A.S, 2005, ‘The human retroviruses’, in Kasper D.L, Faucy A.S., Longo D.L., Braunwald E., Hausler S.L. & Jameson S.L. (eds.), *Harrison’s principles of internal medicine*, 16th edn, pp. 1071–1075, McGraw-Hill, New York.

[CIT0014] Negash T.G, 2014, ‘Review of prevention of mother-to-child transmission of HIV in Addis Ababa, Ethiopia’, DLit et Phil thesis, University of South Africa, Pretoria.

[CIT0015] Okonji J.A., Zeh C., Weidle P.J., Williamson J., Akoth B., Masaba R.O. et al, 2012, ‘CD4, viral load response, and adherence among antiretroviral-naïve breast-feeding women receiving triple antiretroviral prophylaxis for prevention of mother to child transmission of HIV in Kisumu, Kenya’, *Journal of Acquired Immune Deficiency Syndromes* 61(2), 249–257. 10.1097/QAI.0b013e318262514f 22692094

[CIT0016] Sayed A.R, 2007, *Epidemiology. A research manual for South Africa*, 2nd edn, ABC Press, Cape Town.

[CIT0017] World Health Organization, 2005, *Interim WHO clinical staging of HIV/AIDS and HIV/AIDS case definitions for surveillance*, viewed 10 September 2009, from: http://www.who.int/hiv/pub/guidelines/casedefinitions/en

[CIT0018] World Health Organization, 2010, *Guidelines on HIV and infant feeding*, viewed 27 November 2012, from http://apps.who.int/iris/bitstream/10665/44345/1/9789241599535_eng.pdf

[CIT0019] World Health Organization, 2011, *Towards the elimination of mother-to-child transmission of HIV*, viewed 07 April 2012, from http://www.who.int

[CIT0020] World Health Organization, 2013, *Consolidated guidelines on the use of antiretroviral drugs for treating and preventing HIV infection*, viewed 12 July 2013, from http://www.who.int/hiv/pub/guidelines/arv2013/en/index.html 24716260

